# Case study: a community-led outreach programme to improve eye care service delivery in Sri Lanka

**Published:** 2022-09-20

**Authors:** Asela Abeydeera

**Affiliations:** President, Association of Community Ophthalmologists of Sri Lanka (SLACO).

**A community in Sri Lanka got together to ask for, co-fund, and co-ordinate the eye care services they needed.**

The latest national blindness survey conducted in Sri Lanka has estimated that there are over 100,000 adults over the age of 40 in the country who are blind due to unoperated cataract.^1^ However, access to eye care is limited as more than 80% of the population lives in rural areas^2^; the COVID-19 pandemic has further restricted access.

**Figure:**
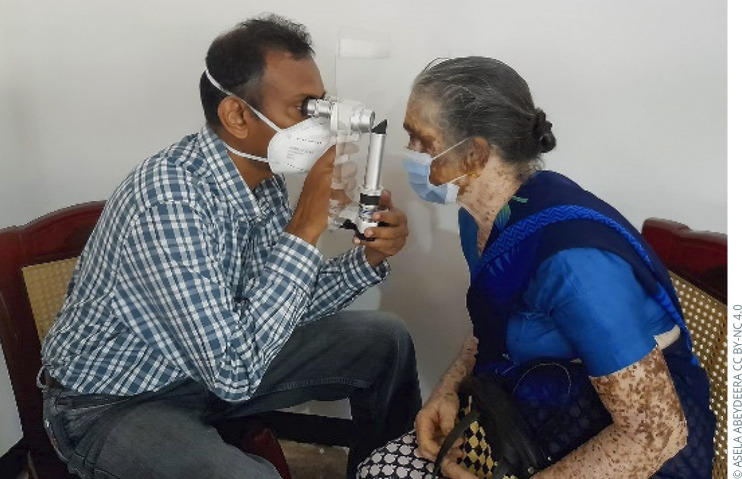
Examining a patient using a hand-held slit lamp. sri lanka

To address the situation, the Association of Community Ophthalmologists of Sri Lanka (SLACO) has been carrying out regular, free outreach eye care visits in remote areas for the last five to six years. The main purpose of these visits are to offer free reading spectacles and refer patients for free cataract surgical services. SLACO members give their time free of charge.

Before SLACO will agree to carry out an outreach visit, the following criteria must be met:

- The area must be more than 20–25 km from the nearest secondary eye unit.
- A local or community organisation must be willing to organise and promote the event.
- There must be funding to cover the local costs of hosting the event, such as refreshments and utility bills. SLACO relies on donations to cover the cost of travel, surgery, medicines, and follow-up examinations.

**Figure:**
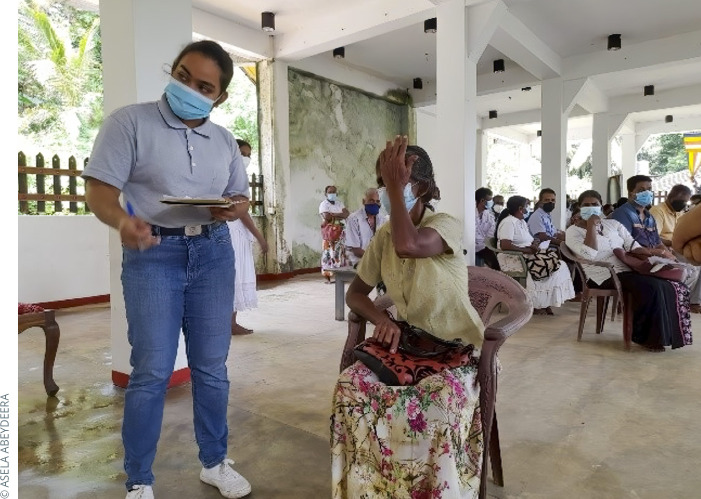
Patients waiting for cataract surgery. sri lanka

## Example: Niunhalla village

An outreach visit to the village of Niunhalla, 40 km from the capital Colombo, demonstrates how the community joined forces to ask for, co-fund, and co-ordinate the eye care services they needed.

- A local organisation for older people in the village of Niunhalla, 23 km away from the nearest secondary eye unit, recognised the need for eye care in their local community and contacted SLACO to request an outreach visit. They also promoted the event and the village leader supervised pre-registration.
- A local philanthropist offered to pay some of the costs of providing near vision spectacles and cataract surgery.
- The village leader (known as grama niladhari) offered to coordinate the event.
- The chief monk of the local Buddhist temple provided a large hall in the temple where eye examinations could be carried out.
- Tzu-Chi Foundation Sri Lanka, a Taiwan-based international Buddhist charity organisation, offered free transport for the team and practial support during the visit: volunteer members from the charity were trained to register patients on arrival and to assess their visual acuity.

Numbers were limited to 125, on a first-come, first-served basis, in accordance with the restrictions issued by the health authorities to minimise the spread of COVID-19. Community members who registered were given different arrival times to avoid overcrowding and comply with COVID-19 safety measures.

SLACO chose to host the event on a public holiday in order to improve participation and make it as convenient as possible for everyone involved.

### On the day

After registration, distance and near visual acuity of patients was assessed and their eyes were examined. The community ophthalmologist from SLACO examined 143 people, as walk-in patients were permitted, and 120 people received near-vision spectacles.

Forty-three people had operable cataract and were given pre-operative assessments on the day (ECG, fasting blood sugar, and a blood pressure check). SLACO arranged for them to be taken to a charity eye hospital in Colombo as the three nearest government eye units had a two-year waiting list.

**Figure:**
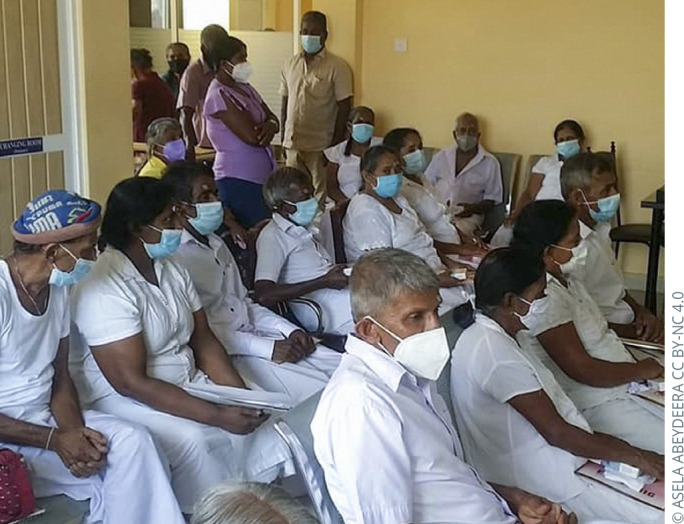
Patients waiting for cataract surgery. sri lanka

### Cataract surgery

The cataract patients were transported to the hospital for day surgery and received free pre- and postoperative eye drops and meals.

Postoperative examinations were conducted by a member of SLACO who visited the village one day, seven days, and thirty days surgery. Thereafter, patients were discharged from follow-up.

**Figure:**
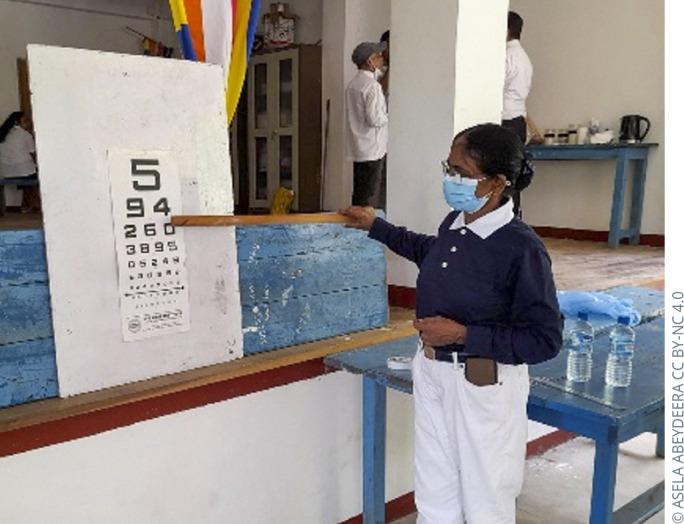
A member of Tzu Chi Foundation assessing the visual acuity of a participant. sri lanka
